# Reasons for stopping Pressurized IntraPeritoneal Aerosol Chemotherapy (PIPAC): A retrospective study to improve future patient selection

**DOI:** 10.1371/journal.pone.0287785

**Published:** 2023-11-30

**Authors:** Anne-Cécile Ezanno, Brice Malgras, Pierre-Louis Conan, Adeline Aime, Jade Fawaz, Hugo Picchi, Solène Doat, Marc Pocard

**Affiliations:** 1 Department of Digestive Surgery, Begin Military Teaching Hospital, Saint Mandé, France; 2 French Military Health Service Academy, Ecole du Val de Grâce, Paris, France; 3 Department of Infectiology, Begin Military Teaching Hospital, Saint Mandé, France; 4 Department of Oncology, Begin Military Teaching Hospital, Saint Mandé, France; 5 Department of Hepato Gastro Enterology, La Pitié Salpétrière Hospital, Paris, France; 6 Department of Digestive Surgery, La Pitié Salpetrière Hospital, Paris, France; 7 INSERM, U965 CART Unit, Paris, France; Morgagni-Pierantoni Hospital, ITALY

## Abstract

To improve the prognosis and maintain quality of life in patients with peritoneal metastasis (PM), a novel treatment has been introduced–pressurized intraperitoneal aerosol chemotherapy (PIPAC). The majority of teams propose at least 3 PIPAC procedures. However, for many patients PIPAC is stopped after only one or two procedures. The aim of this study was to identify the reasons for stopping PIPAC after only one or two procedures and to establish a profile of poor candidates. This retrospective, multicenter cohort study included all patients who underwent PIPAC in three French expert centers between 2015 and 2021. A total of 268 PIPAC procedures were performed in 89 patients. Of them, 48.3% of patients underwent fewer than three procedures: 28.1% had one, 20.2% two and 51.7% three or more PIPAC procedures. The main reason for stopping PIPAC, regardless of the number of procedures, was disease progression, in 55.8% of cases. Other reasons for stopping PIPAC were non-access to the abdominal cavity (7.9%), conversion to cytoreductive surgery (13.5%), post-PIPAC adverse events (7.9%), patients’ wishes (10.1%) and death (2.2%). In univariate analysis, patients who received fewer than three PIPACs less frequently had chemotherapy beforehand (91% vs 100%, *p = 0*.*05*), less frequently had bimodal treatment (70% vs 87%, *p = 0*.*04*), had more ascites (median 80 ml vs 50 ml, *p = 0*.*05*) and more frequently had carcinomatosic ascites (48.8% vs 23.9%, *p < 0*.*01*). Performing PIPAC alone in chemotherapy-naïve patients with ascites should be avoided.

## Introduction

Peritoneal metastases (PM) are a common evolution of abdominal cancers and are associated with a poor prognosis despite systemic palliative therapy. The need to improve patients’ prognosis and maintain quality of life has prompted research efforts to develop new treatment alternatives. Pressurized intraperitoneal aerosol chemotherapy (PIPAC) has emerged in the past decade as a novel method of drug delivery with encouraging results in the treatment of PM from various primary tumors [1–4]. It is an alternative for patients who are not eligible for CRS and HIPEC. The most common indications are PM from ovarian, gastric or colorectal cancer and patients with peritoneal mesothelioma [1]. Usually, at least three PIPAC procedures are proposed at 6±2-week intervals [1, 5], as monotherapy or in combination with systemic chemotherapy [6–8]. Two regimens of intraperitoneal drugs are usually delivered through PIPAC: cisplatin with doxorubicin (C/D) or oxaliplatin as monotherapy. Other drugs (Mitomycin C or Nabpaclitaxel) can be used if necessary [9]. Several studies have reported on the feasibility, tolerance and efficacy of PIPAC and thus have encouraged the widespread use of PIPAC as a novel drug delivery technique [2].

Although repeated PIPAC procedures are feasible in most patients, some studies have reported consistent failure rates following the second PIPAC procedure (>15%) [10]. Thus, a number of patients do not complete the planned three PIPAC procedures [6]. It could be postulated that in the case of non-realization of the full PIPAC course, the benefits to the patient could be limited.

The aim of the present study was to perform a post-hoc analysis of the initial experience of PIPAC for the management of PM. in three tertiary referral centers. The three centers are in Paris, France and for over 5 years were the only ones to perform PIPAC in the region. All senior surgeons know each other and shared indications with the same multidisciplinary tumor management board for the majority of the cases reported. For all these reasons, we considered the homogeneity of the treatment strategy as a solid rationale for analyzing the cases in a pooled analysis. This study was conducted to analyze the reasons for discontinuing a planned course of PIPAC and to establish the profile of poor candidates for PIPAC treatment.

## Materials and methods

### Patient selection

This was a multicenter, retrospective study including all patients who received PIPAC between December 2015 and September 2021 for non-resectable PM in three French hospitals experienced in PM management (Lariboisière University Hospital, Begin Military hospital and Pitié Salpêtrière University Hospital). The study was approved by the Ethics Committee of the coordinating center *(Research Office of the Training*, *Research and Innovation Directorate & Scientific Council of the Bégin Military Teaching Hospital)* in accordance with the ethical standards of the Helsinki Declaration of 1975. Each patient was asked to give written informed consent for data collection, as well as for publication of their de-identified data.

Before treatment, every case was presented to the multidisciplinary tumor board. A PIPAC procedure was only considered for patients with non-resectable PM and without a WHO performance status <2, intestinal obstruction, need for parenteral nutrition, extra-peritoneal disease or allergy to platinum compounds or doxorubicin. For each patient, the tumor board validated the PIPAC procedure. Some patients had been treated in the context of a prospective randomized trial (EstoK 01 [11] or PIPOX [12]).

For each patient who received at least one PIPAC procedure, the following data were extracted: sex, body mass index (BMI), age at the time of the first procedure, origin of PM, history of chemotherapy prior to PIPAC. During PIPAC procedure, ascites (presence and volume) was assessed and the extent of PM rated according to the Peritoneal Cancer Index (PCI). For each patient, the number of performed PIPAC procedures and reason for discontinuing PIPAC were recorded. Post-surgical/PIPAC complications were collected according to the Common Terminology Criteria for Adverse Events (CTCAE- Version 5.0). Only major complications CTCAE grade 3–4 were analyzed.

### PIPAC surgical technique

The technique, safety protocol and treatment regimens are highly standardized among PIPAC referral centers, as described in other studies [1]. Chemotherapy agents considered for PIPAC administration were the combination of cisplatin 7.5 mg/m^2^ in 150 ml NaCl solution and doxorubicin 1.5 mg/m^2^ in 50 ml NaCl solution or oxaliplatin 92 mg/m^2^ in 200 ml of 5% glucose solution. We note that since the publication of Hübner *et al*., new doses of cisplatin and doxorubicin have been in use, respectively 10.5 and 2.1 mg/m^2^ [13]. Once the chemotherapy has been fully injected, the capnoperitoneum is maintained at a pressure of 12 mmHg for 30 minutes. At the end of the procedure the toxic aerosol is removed through a closed and filtered system to avoid the risk of exposure. Then, the ports are removed and the wounds are closed [14].

### Statistical analysis

Demographic, surgical and oncologic details for all patients were prospectively entered into a computerized, coded database designed specifically for quality control of the PIPAC cohort. Microsoft Excel version 16.60 was used for data collection. Continuous data are presented using descriptive statistics (mean ± standard deviation or median [25^th^–75^th^ percentiles; Q1–Q3). Qualitative data are presented using frequencies and percentages. Quantitative parameters were compared between groups using the Student’s t-test or Mann-Whitney test when normality was rejected. Qualitative parameters were compared between groups using the chi-square test or Fisher’s exact test, as appropriate. A threshold of 5% was used to define the significance of the statistical tests. We performed only univariate analysis, for reasons of statistical methodology the number of patients in some groups was too low. Statistical analysis was performed using R statistical analysis software for scientific and medical publications.

## Results

A total of 96 patients were eligible for PIPAC after discussion by the multidisciplinary tumor board. However, for seven of the patients no abdominal access was possible (7.3%). In total, 268 PIPAC procedures were performed in 89 patients (48 female (54%), 41 male (46%)) with a median age of 63 years. The origins of the PM were gastric, colorectal, mesothelioma, ovarian, biliopancreatic or other in 40 (44.9%), 25 (28.1%), 5 (5.7%), 7 (7.9%), 6 (6.7%) and 6 (6.7%) patients, respectively. In 85 patients (96%), systemic chemotherapy was performed before and between PIPAC sessions with a median of 12 cycles, and 32 patients (38%) underwent more than two lines of preoperative chemotherapy. The median time interval from diagnosis of PM to the first PIPAC procedure was 7 months (min 0-max 178 months, Q1 = 4, Q3 = 13 months). In 25 patients (28%), a previous laparotomy had been performed and 29 (32.6%) had Hyperthermic IntraPEritoneal Chemotherapy (HIPEC) procedures (Table 1). At the time of the first PIPAC procedure the median PCI was 19.5 (2.8–31.0), and 52 of the 89 patients (58.4%) had ascites.

**Table 1:** Demographic characteristics of patients eligible for pressurized intraperitoneal aerosol chemotherapy for peritoneal metastases.

*Parameter*		*Patients eligible for PIPAC (n = 96)*	*Patients in whom PIPAC was performed (n = 89)*	*PP cohort*		*P value*
				<3 PIPAC procedures n = 43	≥3 PIPAC procedures n = 46	
*Median Age (Q25–75)*		62 (50–67)	63 (53–67)	64(52; 69)	62 (53–66)	0.49
*Sex*, *n (%)*	Male	43 (44.8%)	41 (46%)	21 (49%)	20 (43%)	0.61
	Female	53 (55.2%)	48 (54%)	22 (51%)	26 (57%)	
*Median BMI (kg/m* ^*2*^ *)*		23.5 (21; 25.6)	23.6 (21–26.2)	24 (4.60)	23.4 (3.46)	0.46
*ASA*, *n (%)*	1	7 (7.4%)	7 (7.9%)	4 (9.3%)	3 (6.7%)	0.94
	2	71 (74.7%)	65 (73.9%)	31 (72%)	34 (76%)	
	3	17 (17.9%)	16 (18.2%)	8 (19%)	8 (18%)	
*Origin of PC*, *n (%)*	Gastric	43 (44.8%)	40 (44.9%)	16 (37%)	24 (52%)	0.55
	Colorectal	28 (29.2%)	25 (28.1%)	13 (30%)	12 (26%)	
	Ovarian	6 (6.25%)	5 (5.7%)	3 (7%)	2 (4.3%)	
	Mesothelioma	7 (7.3%)	7 (7.9%)	4 (9.3%)	3 (6.5%)	
	Bilio-pancreatic	6 (6.25%)	6 (6.7%)	4 (9.3%)	2 (4.3%)	
	Other	6 (6.25%)	6 (6.7%)	3 (7%)	3 (6.5%)	
*Synchronous PC*, *n (%)*	No	25 (26.1%)	23 (25.8%)	11 (25.6%)	12 (26.1%)	0.96
	Yes	71 (73.9%)	66 (74.2%)	32 (74.4%)	34 (73.9%)	
*Previous laparotomy*, *n (%)*		53 (55%)	25 (28%)	28 (65%)	36 (78%)	0.17
*Previous CRS + HIPEC*, *n (%)*		31 (32%)	29 (32.6%)	15 (34.8%)	14 (30.4%)	0.65
*Preoperative Chemotherapy*, *n (%)*		92 (95.8%)	85 (96%)	39 (91%)	46 (100%)	**0.05**
*≥12 cycles*, *n (%)*		51 (53.1%)	48 (53.9%)	25 (58%)	23 (50%)	0.44
*≥3 lines*,*n (%)*		31 (32%)	32 (38%)	18 (42%)	11 (24%)	0.07
*Number of chemo cycles/patient (median*, *Q25–75)*		12(7–17)	12 (7–17)	13.8 (9.4)	12.2 (6.44)	0.35
*Duration of carcinosis (months)*, *median*, *Q25–75*		8(4.00–13.0)	7(4–13)	6(3 16.0)	7 (4; 11)	0.62

CRS = CytoReductive Surgery, HIPEC = Hyperthermic IntraPEritoneal Chemotherapy; BMI = body mass index; PIPAC = Pressurized IntraPeritoneal Aerosol Chemotherapy

**Fig 1:**
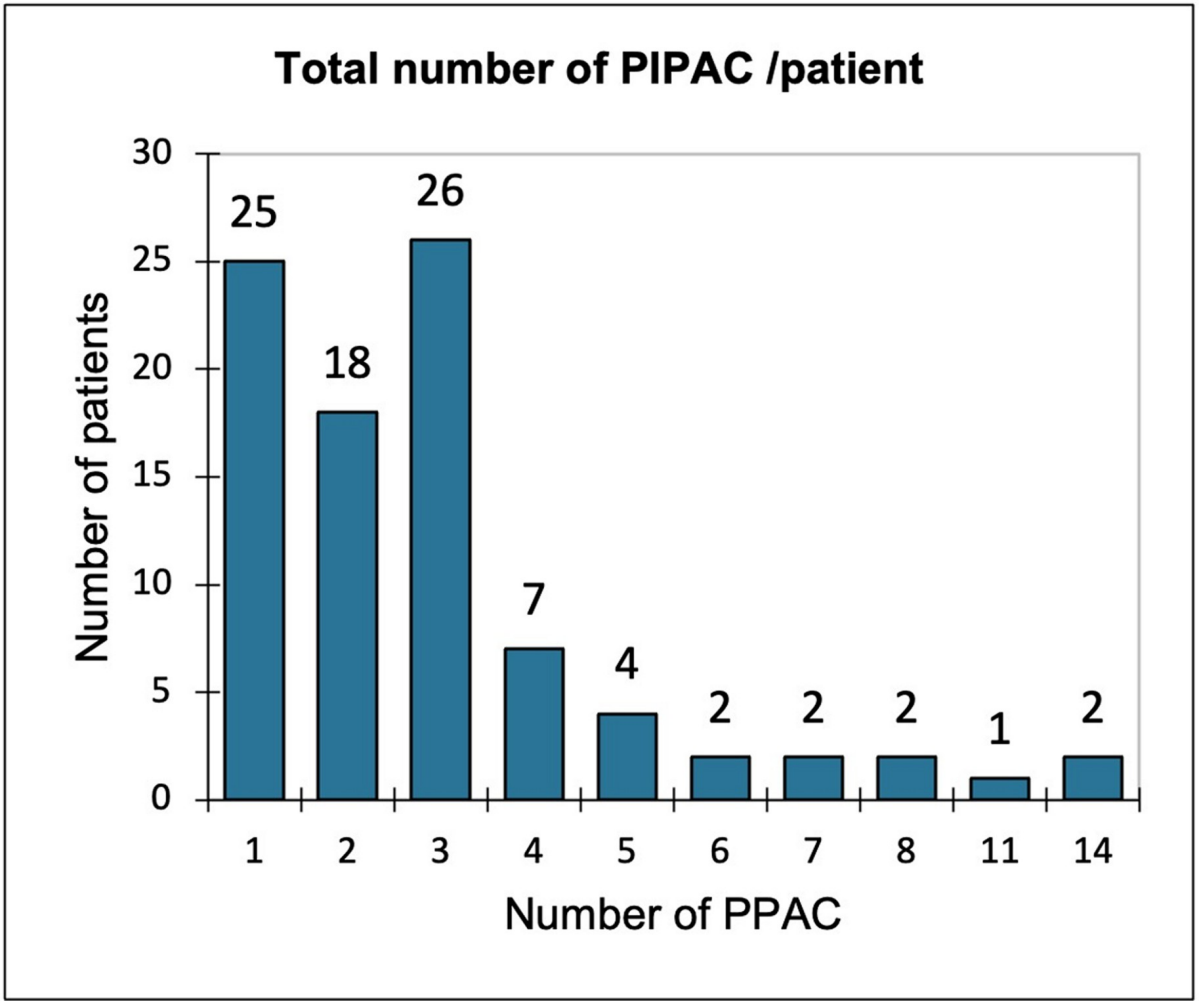
Number of PIPAC per patient.

**Fig 2:**
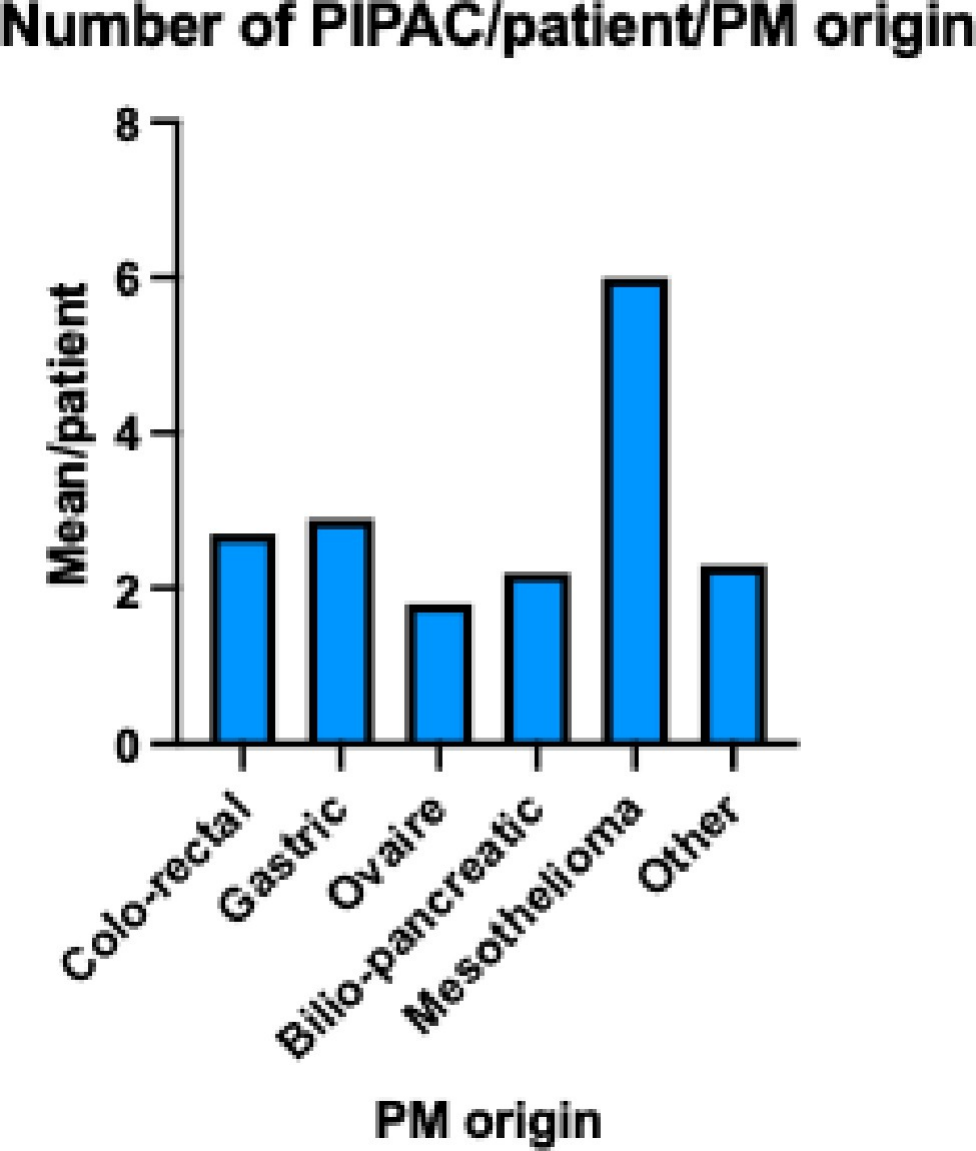
Mean number of PIPAC procedures according to origin of PM.

Forty-six patients (51.7%) received more than two PIPAC procedures: 25 (28.1%) had one PIPAC, 18 had two PIPAC (20.2%), 26 patients (29.2%) completed the planned three procedures, and 20 (22.5%) patients had four or more PIPAC procedures (Figs 1 and 2). Two patients with mesothelioma underwent 14 PIPAC procedures, and another procedure is planned for one.

Subgroup analysis indicated that patients who underwent only 2 PIPAC procedures or less, had a significantly higher proportion of high-volume ascites (mean 1116 ± 2137 ml vs 474 ± 1559 ml; *p = 0*.*05*) and malignant ascites (48.8%, vs 23.9%; *p <0*.*01*), a lower proportion of dual treatment (PIPAC and systemic chemotherapy) (70% vs 87%, *p = 0*.*04*) and less preoperative chemotherapy (91% vs 100%; *p = 0*.*05*). Patients who received only one PIPAC procedure did not show a higher PCI score (19 vs 20, *p = 0*.*81*) or higher proportion of ascites (65.1% vs 52.2%; p = 0.22), and there was no significant association between an aggressive pathology with a past history and first PIPAC (mean 15.4 ± 28.1 vs 9.87 ± 10.8; p = 0.63) (Table 2).

**Table 2:** Intra-operative characteristics of pressurized intraperitoneal aerosol chemotherapy.

PARAMETER		ALL		*<3 PIPAC*		*≥3 PIPAC*		*P* VALUE
		N = 89		*N = 43*		*N = 46*		
		n (%)	Median (Q25–Q75)	n (%)	Median (Q25–Q75)	n (%)	Median (Q25–Q75)	
**IMPOSSIBLE ABDOMINAL ACCESS**		7 (7.9%)		5 (11.6%)		**2 (4.3%)**		-
**PIPAC SESSIONS PER PATIENT MEDIAN (Q25–Q75)**			3 (1–3)		1 (1–2)		3 (3–5)	-
**PIPAC SESSIONS**	1	-		25 (58.1%)		-		
	2	-		18 (41.9%)		-		
	3					26 (56.5%)		
	>4					20 (43.5%)		
**BIMODAL TREATMENT, N (%)**		70 (78.6%)		30 (70%)		40 (87%)		**0.04**
**CHEMO REGIMEN**	Cisplatin+doxorubicin	42 (47.2%)		24 (55.8%)		21 (46%)		0.69
	Oxaliplatin	45 (50.6%)		18 (41.8%)		24 (52%)		
**PCI AT TIME OF FIRST PIPAC, MEDIAN (Q25–Q75)**			19.5 (12.8; 31)		19(12; 32.5)		20(14.2; 30.8)	0.81
**PATIENTS WITH ASCITES (/%)**		52 (58.4%)		28 (65.1%)		24 (52.2%)		0.22
**ASCITES VOLUME (ML, MEDIAN, Q25–Q75)**			50 (0; 200)		50 (0; 1100)		80(50; 250)	**0.05**
**CYTOLOGY POSITIVE, N (%)**		32 (36%)		21 (48.8%)		11 (23.9%)		**<0.01**

**Fig 3:**
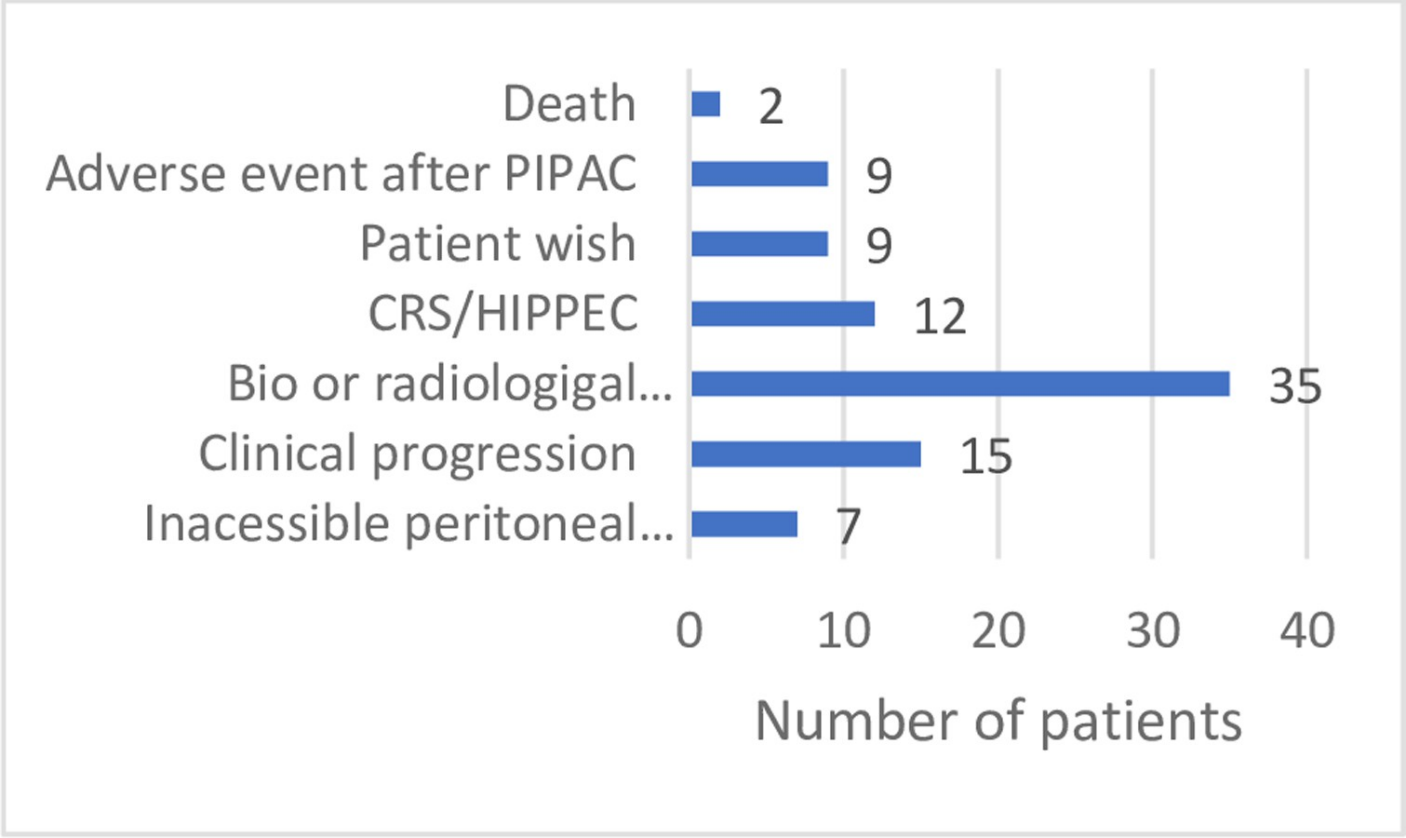
Reasons for stopping PIPAC. * PIPAC = Pressurized IntraPeritoneal Aerosol Chemotherapy. CRS/HIPEC = CytoReductive Surgery/Hyperthermic IntraPeritoneal Chemotherapy.

For 50 patients (56.2%) the main reason for discontinuing the planned PIPAC course was clinical or biological-radiological progression of PM. In seven patients, minimally invasive access to the abdominal cavity was no longer technically feasible (secondary non-access rate of 7.9%). Other reasons for stopping PIPAC, regardless of the number of procedures, were conversion to curative intent cytoreductive surgery (CRS) and HIPEC 13.5% (n = 12), the patient’s wish in 10.1% (n = 9), an adverse event in 10.1% (n = 9) and death in 2.2% (Fig 3). In our cohort, conversion to curative intent CRS or HIPEC was possible in 13.5% (n = 12) of cases: 2 HIPEC were performed for gastric tumors, one CRS for ovarian cancer (HIPEC indicated but not performed because of a major lesion), one HIPEC for mesothelioma, and 3 HIPEC for colorectal cancer.

Of the 89 patients, 43 received <3 PIPAC (48.3%) and 46 ≥3 PIPAC (51.7%) procedures. In a subgroup analysis of patients who received fewer than three PIPAC procedures, the main reason for discontinuing PIPAC before the planned third procedure was disease progression in 55.9%. Other reasons were conversion to curative intent CRS and HIPEC (7%) or adverse events / PIPAC complications (16.3%) (intra-operative bowel injury, bowel obstruction, bleeding or healing difficulty). Two patients (4.6%) had premature interruption of PIPAC therapy due to the patient’s wish. However, the only significant reason of discontinuing PIPAC before the third recommended procedure was an adverse event (16.3% vs 4.3%, *p = 0*.*05*). Moreover, biological or radiological progression and adverse events were the two main reasons of discontinuing PIPAC after only one PIPAC procedure (respectively, *p = 0*.*02* and *p = 0*.*01*). Biological or radiological progression was another significant reason for discontinuing PIPAC after two procedures (*p < 0*.*01*) (Table 3).

**Table 3:** Analysis of incompletion of the planned course of treatment.

Reason for stopping PIPAC treatment	*All n; %*	*PIPAC = 1*	*P*	*PIPAC = 2*	*P*	*PIPAC < 3*	*PIPAC ≥ 3*		*P value*
Number of procedures	89 (100%)	25 (28.1%)		18 (20%)		43 (48.3%)		46 (51.7%)	-
*Inaccessible peritoneal cavity*	7 (7.9%)	4 (16%)	*0*.*09*	1 (5.6%)	1	5 (11.6%)	2 (4.3%)		0.26
*CRS/HIPEC*	12 (13 .5%)	2 (8%)	*0*.*5*	1 (5.6%)	0.45	3 (7%)	9 (19.6%)		0.08
*Clinical progression*	15 (16.8%)	5 (20%)	*0*.*75*	3 (16.6%)	1	7 (16.3%)	8 (17.4%)		0.06
*Bio or radiological progression*	35 (39%)	5 (20%)	***0*.*02***	12 (66.6%)	**<0.01**	17 (39.6%)	18 (39.1%)		0.97
*Patient’s wish*	9 (10.1%)	2 (8%)	*1*	0	0.19	2 (4.6%)	7 (15.3%)		0.16
*Adverse event after least one PIPAC*	9 (10.1%)	5 (20%)	***0*.*01***	2 (11.1%)	1	7 (16.3%)	2 (4.3%)		**0.05**
*Death*	2 (2.2%)	2 (2.2%)	*0*.*07*	0	1	2(4.6%)	0		0.23

## Discussion

The results of this retrospective, multicenter study suggest that PIPAC without prior chemotherapy, or in the presence of malignant ascites is associated with a high risk of discontinuing PIPAC after one or two procedures. These points appear important and should be considered in the selection of patients with PM for PIPAC treatment. Adverse events are also often the cause of stopping PIPAC treatment.

Some patients who are eligible for PIPAC treatment have had prior surgery, CRS or HIPEC, and for these patients peritoneal access for a PIPAC procedure may be more difficult [15, 16]. We observed a rate of 7.3% (n = 7/96) of patients with abdominal access failure during the first PIPAC procedure and 7.9% (n = 7/89) during the second or third procedure. Similar or higher rates of failure to achieve abdominal access have been reported in the literature [17–19]. Indeed, minimally invasive surgery in patients with multiple prior surgical interventions, such as the patients in this cohort, is challenging and associated with high conversion rates [16]. Adhesions, obliteration of the peritoneal space (omental cake or great nodules) and intestinal distension have an impact on access to the abdomen. A first PIPAC procedure, intraperitoneal chemotherapy and repeated PIPAC are well known as inducing peritoneal sclerosis [19, 20], which probably explains the reported secondary non-access rate of 0–35% [21–24]. In our study, we had a non-access rate of 11.6% for the second and subsequent procedures taken together.

In our cohort, patients had a mean of three PIPAC procedures. Among the 89 patients, 48.3% (n = 43) received fewer than three PIPAC procedures, which is similar to the results reported by Balmer et al. [6]. The reasons we found for discontinuation of PIPAC before a third procedure were conversion to CRS/HIPEC, disease progression, the patient’s wish and adverse events. Discontinuation of PIPAC due to disease progression and clinical deterioration raises questions concerning patient selection. For many patients, the absence of proof of efficacy of a second- or third-line systemic treatment leads them to think of PIPAC as highly promising option. It is probably this hope that prompts us to consider this treatment for these patients.

The control of ascites is a potential indication for PIPAC, even though high volumes of ascites may reflect very late stage disease and a high probability of treatment failure [24]. In our study, large ascites volume was a significant factor for discontinuation of PIPAC (p = 0.05). In the presence of high volumes of ascites, the indication should be considered with care. In 2020, Di Giorgio et al. reported that patients with high-volume ascites underwent only one procedure [4]. The presence of high volumes of ascites also has an impact on quality of life (QOL). Nevertheless, several studies have demonstrated that this new therapy is feasible and safe with a very high tolerance [25, 26] and stabilizes or improves patient’s QOL [27]. If ascites is present together with another indication for PIPAC, such as mesothelioma, this could be one reason for treatment failure and a reason to stop. Typically, for mesothelioma, we noted success in some patients with prolonged ascites control, such as the patient who had more than 15 PIPAC procedures. In contrast, for some patients with deterioration in their general status and contraindication to intravenous chemotherapy, a failure to stop ascites with PIPAC alone was observed in the monotherapy group.

In our cohort, conversion to curative intent CRS or HIPEC was possible in 13.5% (n = 12) of cases: 2 HIPEC were performed for gastric tumors, one CRS for ovarian cancer (HIPEC indicated but not performed because of a major lesion), one HIPEC for mesothelioma, and 3 HIPEC for colorectal cancer. These small numbers does not allow us to propose the type of original tumor for which PIPAC would be the most promising, but it suggests its potential usefulness in reducing PM. Since 2011, when PIPAC was introduced for PM, stopping of PIPAC due to conversion to CRS and HIPEC has been frequently cited. A French study reported that the PCI decreased after repeated PIPAC procedures, and up to 15% of patients became candidates for CRS and HIPEC [28]. Recently, PIPAC demonstrated encouraging results in patients with unresectable PM, and Alyami et al. [26] demonstrated that CRS and HIPEC can be achieved in strictly selected patients with unresectable PM at diagnosis after repeated PIPAC procedures. They reported a rate of 14.4% of CRS/HIPEC after a median of three PIPAC procedures. In our study, 13.5% of patients were eligible for CRS/HIPEC before a third PIPAC procedure. In four patients, CRS and HIPEC were proposed after the first PIPAC. For these patients, the indication for PIPAC might have been unnecessary and perhaps they could have undergone CRS and HIPEC directly.

Post-operative morbidity is another reason for discontinuing PIPAC. Our post-operative morbidity rate was 11.2% (n = 10). The most common intraoperative complication was iatrogenic bowel injury (0–3% of total PIPAC procedures) [10]. Winkler et al. [27] also found that bowel injury related to Veress needle or trocar insertion was the most common complication [29]. For this reason, research is needed into improving methods of access to the peritoneal cavity to avoid such complications [15, 29]. A review found that post-operative mortality varied from 0 to 2% of total PIPAC procedures [10] and was mainly caused by unrecognized bowel injury or bowel obstruction. This was the cause of mortality for two patients in our cohort, in line with the review.

In our cohort, another reason for patients having fewer than three procedures was the patient’s wish. Ten patients stopped their PIPAC programs prematurely (10.1%), the reasons being linked to a logistical problem due to the relocation our surgical center. Indeed, between 2016 and 2019, few centers offered this treatment and patients were obliged to travel long distances, leading to discouragement and stopping the treatment.

The main limitations of our study were the heterogeneity of the inclusion criteria and missing data (often the case in retrospective studies) particularly regarding the reason for stopping treatment. We only performed a bivariate analysis because of the heterogeneity in our cohort and thus low sample size for each type of tumor. We included only five cases of mesothelioma, but for this kind of PM etiology the patients had an average of six PIPAC procedures, which may point to some effectiveness of PIPAC for this indication. Our findings need to be validated using larger cohorts.

## Conclusions

In conclusion, the reason for discontinuing PIPAC is often disease progression, which raises questions regarding the eligibility criteria currently used. Bimodal treatment and no malignant ascites increases the chances of completing three or more PIPAC procedures. There is a need for further investigations to improve patient selection.

## Supporting information

S1 Data (XLSX)
