# Correction: Reasons for stopping Pressurized IntraPeritoneal Aerosol Chemotherapy (PIPAC): A retrospective study to improve future patient selection

**DOI:** 10.1371/journal.pone.0335021

**Published:** 2025-10-17

**Authors:** Anne-Cécile Ezanno, Brice Malgras, Pierre-Louis Conan, Adeline Aime, Jade Fawaz, Hugo Picchi, Solène Doat, Marc Pocard

In the Results section, there is an error in the fourth sentence of the first paragraph. The correct sentence is: The origins of the PM were gastric, colorectal, ovarian, mesothelioma, biliopancreatic or other in 40 (44.9%), 25 (28.1%), 5 (5.7%), 7 (7.9%), 6 (6.7%) and 6 (6.7%) patients, respectively.

In the Discussion section, there is an error in the third sentence of the last paragraph. The correct sentence is: We included seven cases of mesothelioma, but for this kind of PM etiology the patients had an average of six PIPAC procedures, which may point to some effectiveness of PIPAC for this indication.
